# Supplementary figures and images for: Implementation status of postoperative rehabilitation for older patients with hip fracture in Kyoto City, Japan: A population-based study using medical and long-term care insurance claims data
Source: PLoS One. 2024 Sep 12;19(9):e0307889. doi: 10.1371/journal.pone.0307889 (PMC11392384; doi:10.1371/journal.pone.0307889)

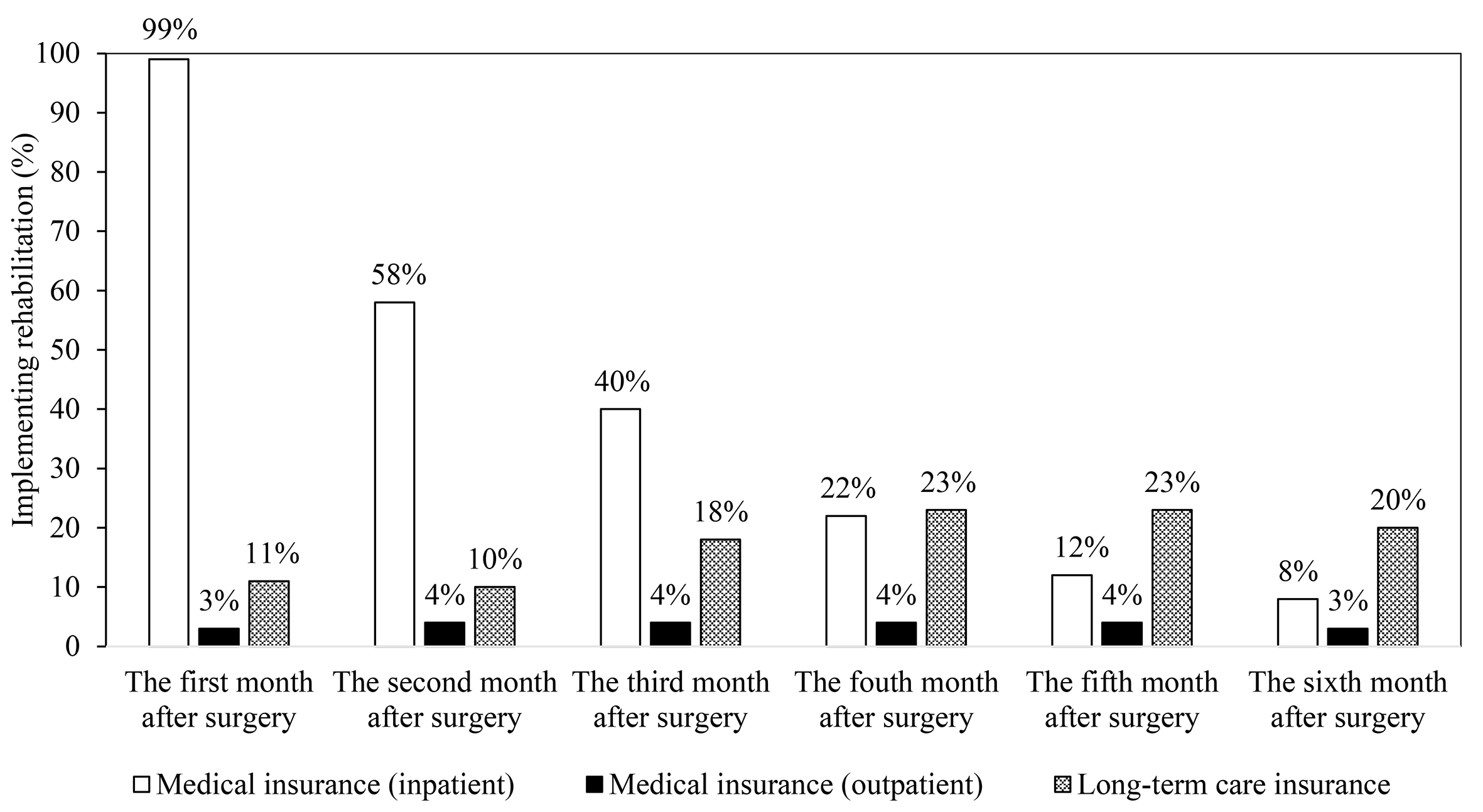

Supplement: S1 Fig — The number of patients in the same month includes those who underwent rehabilitation by medical insurance (inpatient and outpatient) and long-term care insurance. Therefore, per month, the total number of patients in each category may exceed the number in the analysis. (TIF) [file pone.0307889.s001.tif]
