# Supplementary material for: Implementation status of postoperative rehabilitation for older patients with hip fracture in Kyoto City, Japan: A population-based study using medical and long-term care insurance claims data
Source: PLoS One. 2024 Sep 12;19(9):e0307889. doi: 10.1371/journal.pone.0307889 (PMC11392384; doi:10.1371/journal.pone.0307889)
Supplement: Attachment — Submitted filename: Response to Reviewers.docx [file pone.0307889.s008.docx]

July 6, 2024

Dr. Antimo Moretti

Academic Editor

PLOS ONE

Dear Dr. Moretti and Reviewers,

Thank you very much for revising our manuscript entitled “**Implementation status of postoperative rehabilitation for older patients with hip fracture in Kyoto City, Japan: A population-based study using medical and long-term care insurance claims data.**” We have responded to the editor’s and reviewers’ comments in the point-by-point responses below and revised the manuscript accordingly.

We hope our revised manuscript is now suitable for publication in *PLOS ONE*.

Thank you very much for your time and effort in considering our manuscript for publication in *PLOS ONE*.

Yours sincerely,

Kosuke Sasaki

Department of Health Informatics, School of Public Health,

Graduate School of Medicine, Kyoto University.

Yoshida-Konoe-cho, Sakyo-ku,

Kyoto 606-8501, Japan.

TEL: +81-75-753-9477

E-mail: sasaki.kosuke.u73@kyoto-u.jp

**Reviewers' comments:**

**Major comments:**

1. **Page 3, Line 66: Please consider to clarify more details of the study results which supported a minimum requirement of six months rehabilitation after hip fracture surgery in the Japanese guideline. For example, some countries have introduced the reablement interventions which is a multidisciplinary rehabilitation within 3 months after discharge. I am not sure whether all hip fracture patients should take the six months rehabilitation or not, because the intensity of rehabilitation that can be provided in a hospital may not be available or feasible in a nursing home or at home.**

[Response]

Thank you for the suggestion. We are aware that some countries do not require or continue six months of rehabilitation for various reasons, including differences in healthcare systems and resources. However, Japan has a unique system designed to provide comprehensive rehabilitation through a combination of medical and long-term care insurance. It allows for continuous monitoring even at home and provides both medical and nursing care services.

In Japan, the medical and long-term care insurance systems are designed to ensure that patients receive continuous care. This includes home-based services that support the continuation of rehabilitation beyond acute care hospital settings. Therefore, this study investigated the implementation of rehabilitation services over a 6-month period.

We have rewritten the section to provide more clarity on this matter, as follows:

[Detailed revision]

Clinical practice guidelines from nations including Australia [10], the United Kingdom [11], the United States [12,13], Korea [14], and Japan [15,16] endorse early postoperative rehabilitation for managing hip fractures and continuing post-hospitalization. Although the appropriate duration of rehabilitation varies depending on the healthcare system and the patient's condition, there are reports that rehabilitation for 3 to 6 months is necessary after hip fracture surgeries. A randomized controlled trial of older patients with hip fractures found that outpatient rehabilitation using progressive resistance training for six months after discharge significantly improved walking ability and quality of life compared to low-intensity home exercises [17]. A prospective cohort study found that most aspects of the SF-36, except for the physical role behavior subscale, showed almost complete recovery six months after hip fracture surgeries [18]. Such consistent rehabilitation post-hip surgery enhances physical function [19–21] and quality of life [19,20]. The Japanese guidelines specifically recommend a minimum of six months of postoperative rehabilitation [15,16]. In Japan, the average length of hospital stay is decreasing, making rehabilitation after discharge from acute care hospitals increasingly important [22]. In Japan's multifaceted treatment approach, patient discharge trajectories differ, spanning transfers to other facilities and direct-to-home discharge [23–25]. Uninterrupted rehabilitation is crucial, regardless of transitions in the care setting. (pages 4–5, lines 36–54 in the “Manuscript”)

As you have correctly noted, the appropriate length of rehabilitation may vary depending on the healthcare system and the patient's condition. In this study, it was not possible to determine whether all patients with hip fractures should receive six months of rehabilitation. Noting this as a limitation of the study, we also added the following statement:

“Third, the appropriate duration of rehabilitation may vary depending on the healthcare system and the patient's condition, and this study was not able to determine whether all patients with hip fractures should undergo rehabilitation for six months. However, it does highlight the evidence-practice gap in the implementation of rehabilitation following hip fracture surgeries in Japan.” (page 21, lines 344–349)

2. **Page 3, Line 72: Authors mentioned about two previous studies in Japan on the rehabilitation after hip fracture. Please consider the clarification of more details (i.e.,study design, data, limitations etc.) for these studies. Also, authors can mention the other countries’ studies on it. Furthermore, authors are conducting the analysis to identify factors associated with continuity of rehabilitation, so please consider to add what is known in the previous study on it.**

[Response]

Thank you for your valuable comments. As per your suggestion, we have provided more details concerning the two previous studies conducted in Japan—including their designs, data, and limitations. We have also mentioned some related studies from other countries and included information regarding the factors associated with rehabilitation continuity from previous research. Below are the original and revised texts for your review:

[Before]

Recent Japanese research indicated that most patients with hip fracture in Japan's acute hospitals promptly undergo rehabilitation.[23] Another study found that intensive rehabilitation in these institutions for dementia-affected patients post-hip surgery enhanced ADL upon discharge.[24] However, there is limited clarity on the sustained implementation of rehabilitation in the medium to long term following discharge. Hence, the present study aimed to explore the rehabilitation implementation status and identify factors influencing sustained rehabilitation for elderly hip fracture patients post-discharge in Japan, a nation confronting super-aging challenges.

[Detailed revision]

It has been suggested that there are disparities in access to rehabilitation based on patient characteristics. A systematic review describing the equity in randomized controlled trials of rehabilitation interventions following hip fractures revealed that, in > 50% of the 35 trials, potential participants were systematically excluded based on conditions such as residing in nursing homes, cognitive impairment, mobility or functional impairments, age, and ineligibility for surgery [26]. A retrospective cohort study using the census database of all inpatient services provided by public and private hospitals in New South Wales, Australia, reported that older patients, women, those with dementia, those with high frailty risk, and those residing in long-term care facilities were less likely to receive in-hospital rehabilitation [27,28]. Another retrospective cohort study that made use of the Diagnosis Procedure Combination database, which collects inpatient data from over 1500 hospitals across Japan, reported that almost all patients who underwent hip fracture surgeries in acute care hospitals received short-term rehabilitation [29,30]. However, it also reported that those with dementia had fewer opportunities for inpatient rehabilitation after discharge from acute care hospitals [29]. The rate of mid- to long-term extension of rehabilitation after discharge from acute care hospitals for patients following treatments for hip fractures has not been previously reported on, which has raised some concerns surrounding whether sufficient rehabilitation is being provided. Hence, this study aimed to explore the current status of rehabilitation implementation, and identify the factors influencing sustained rehabilitation in older patients with hip fractures post-discharge in Japan—a nation that is currently facing the challenges associated with a rapidly-aging population. (pages 5–6, lines 55–76)

3. **Page 4, lines 90 – 92: Each data has the different periods for data use. These could make us confused, so please consider to write only actual study period after data management (linkage).**

[Response]

We appreciate your attention to the discrepancy in the duration of data usage. We have revised the manuscript to state that the actual study period was from April 2013 to March 2019 after data management and linkage, to provide greater clarity. The study included patients who underwent surgeries for hip fractures between April 2013 and October 2018 with a follow-up period of 6 months. Therefore, the data-use period was extended to March 2019.

[Before]

In this study, we used the following data of the Medical Care System for the Elderly in the Latter Stage of Life, which is the mandatory medical insurance system for people over 75 years old,[26] basic resident registration (fiscal year [FY] 2010 to 2020), medical insurance claims data (FY 2013 to 2018), LTC insurance claims data (FY 2013 to 2018), and LTC needs certification data (FY 2009 to 2018).

[Detailed revision]

In this study, we used data spanning April 2013 to March 2019 from the following sources: the Japanese “Medical Care System for the Elderly in the Latter Stage of Life” (which is the mandatory medical insurance system for individuals > 75 years of age) [32], basic resident registration, medical insurance claims data, LTC insurance claims data, and LTC needs certification data. These data were merged individually using a unique identifier by Kyoto City and anonymized before we received the data. (page 6, lines 84–90)

4. **Page 4, lines 95 – 97: What is the proportion to what?**

[Response]

This shows the percentage of data to which personal numbers are assigned in the Kyoto City database according to the data type (e.g., medical and long-term care insurance claims). We have revised the wording to more clearly indicate the percentage of each type of data to which personal identification numbers were assigned.

[Before]

The proportions of personal identification numbers assigned in this database were as follows. The proportion of injury and disease codes of the medical insurance claims data was approximately 99%, the medical care activities codes were about 99%, the LTC insurance claims were about 98%, and the LTC needs certification data was about 87%.

[Detailed revision]

Personal identification numbers were assigned to ~99% of the medical claims data for injury and illness codes (385,109,527 of 387,547,567 records), ~99% of the medical activity codes (1,237,350,713 of 1,253,415,997 records), ~98% of the LTC insurance claims data (52,387,340 of 53,494,825 records), and ~89% of the LTC care-requiring certification data (861,376 of 964,429 records). (page 6, lines 91–95)

5. **Page 6, line 126: Please explain the Japanese system of rehabilitation between medical and long-term care insurance. We are not sure why authors must use the both of them.**

[Response]

Thank you for your comments. In Japan, medical and LTC insurance are distinct systems; therefore, claims are filed separately. Therefore, previous studies typically examined either medical or LTC insurance claims in isolation. However, a major strength of our study lies in its integration of medical and LTC insurance claims to provide a comprehensive view of the rehabilitation process. We have added a description of the Japanese medical and long-term care insurance rehabilitation systems to further clarify this.

[Detailed revision]

The primary outcome was the implementation of rehabilitation for six months after hip fracture surgery as a process measure. We defined the use of rehabilitation as rehabilitation-related claims filed under medical or LTC insurance at least once per month following hip fracture surgery (Tables S2 and S3). In Japan, there are two types of insurance-covered rehabilitation: medical and LTC. Generally, acute and restorative rehabilitation is covered by medical insurance, while chronic rehabilitation is covered by both insurance types. This distinction ensures that patients receive appropriate care tailored to their stage of recovery and long-term needs [24]. To use LTC insurance services, patients must be certified based on their physical and cognitive functions, as well as their care and medical needs. Once certified, patients are required to use LTC insurance rehabilitation services, except during hospitalization or the initial recovery phase [34]. Because this study aimed to investigate the implementation of mid- to long-term rehabilitation after hip fractures, we included both medical and long-term care insurance data. The definition of implementing rehabilitation was defined based on the advice of a physiatrist (MT), physical therapists (KS and HU), and an occupational therapist (TO). (page 8, lines 122–137)

6. **Table S3: Some residents in special nursing home for the elderly (Toku-you in Japanese) have received the rehabilitation. Is it already included in Table S3?**

[Response]

Thank you for your comments. Unfortunately, our study did not have sufficient data to clarify the implementation status of rehabilitation in specialized nursing homes for older individuals (Toku-you). As was pointed out in Major Comment 15, the implementation of rehabilitation may be influenced by factors related to hospitals and facilities. However, although our database does contain information at both the hospital and facility levels, it has not yet been compiled and maintained in a way that allows for such analyses—thus making it impractical for us to conduct an analysis that considers the nested structure of the population. In response to Major Comment 15, we have added the following text as a limitation of our study.

[Detailed revision]

“Second, this database does not contain information about the physicians who prescribed rehabilitation and information concerning the hospitals or facilities where the patients stayed is not well organized. Therefore, this study could not clarify the relationship between hospitals, facilities, and physicians with the implementation of rehabilitation. Further research is warranted to explore the perspectives of patients and their families on this matter [61,62], as well as facility-level [63] and regional-level factors [64].” (page 21, lines 337–343)

7. **Page 6, lines 132-133: Could you clarify the differences between the comorbidities (i.e., dementia and so on) and 20 types of diseases? What is the purpose of describing the 20 types of diseases?**

[Response]

Thank you for your valuable comments. Here, we describe the 20 types of diseases specified by the Japanese Ministry of Health, Labor, and Welfare. If a patient has a specified illness, home-visit nursing care for intractable diseases can be used ≥ 4 days per week, 2–3 times per day. If a patient applies for long-term care insurance and is certified as approved for support or long-term care, if the illness specified by the Minister of Health, Labor and Welfare falls under this category the patient will be eligible for home-visit nursing care covered by medical insurance and can use home-visit nursing care 2–3 times per day for ≥ 4 per week. If the patient falls into one of the 20 disease type categories specified by Japan's Ministry of Health, Labor, and Welfare, some rehabilitation claims are made on paper. In such cases, some rehabilitation claims may not have been captured by the database used in this study. To clarify that this study may therefore have undercounted rehabilitation practices, we have revised the wording as follows, in the limitations section.

“Fifth, if a patient had any of the 20 disease types specified by the Japanese Ministry of Health, Welfare and Labor, some rehabilitation claims were made on paper and may therefore not have been captured in the database that was used for this study” (pages 21–22, lines 349–352)

In addition, the following statement was added to the Methods section of the manuscript to clarify the reason for specifically identifying the 20 disease types.

“If one or more of the 20 specified disease types are applicable, a patient’s rehabilitation status may not be confirmed by this database, because claims related to some types of rehabilitation are made on paper. We therefore specifically identified these 20 disease types to account for the possibility of underestimating the implementation of rehabilitation.” (page 9, lines 151–155)

8. **Page 6, Lines 139 – 143: Why did authors set the different periods after the surgery to identify the dementia, depression and the frailty risk score, compared to delirium and 20 types of diseases?**

[Response]

Thank you for your comments. For dementia, depression, and frailty scores, we considered the condition up until the time of surgery, to reflect the condition it was conducted. These comorbidities affect the implementation of rehabilitation after hip fracture surgery,^2^ and are necessary to accurately assess a patient’s baseline condition.

On the other hand, delirium and the 20 specific disease types were identified during the postoperative period, to reflect acute conditions and most recent health status. Delirium after elective surgery in older patients has been reported to be a factor in hospital readmission and discharge to other institutions. Therefore, as was done in similar previous studies, delirium was identified in the postoperative setting.^3^

As was noted in Major Comment 8, if one or more of the 20 diseases are applicable, the rehabilitation implementation status may be underestimated, because some claims related to rehabilitation are not included in the database. Therefore, we identified postoperative conditions and evaluated whether any of the 20 specified diseases were applicable to the assessment of rehabilitation implementation status at six months postoperatively.

2. Mitchell R, Draper B, Brodaty H, Close J, Ting HP, Lystad R, et al. An 11-year review of hip fracture hospitalisations, health outcomes, and predictors of access to in-hospital rehabilitation for adults ≥ 65 years living with and without dementia: a population-based cohort study. Osteoporos Int. 2020;31: 465–474. doi:10.1007/s00198-019-05260-8

3. Gleason LJ, Schmitt EM, Kosar CM, Tabloski P, Saczynski JS, Robinson T, et al. Effect of Delirium and Other Major Complications on Outcomes After Elective Surgery in Older Adults. JAMA Surg. 2015;150: 1134–1140. doi:10.1001/jamasurg.2015.2606

9. **Page 7, line 153: I am not sure what the convalescent rehabilitation ward is. Please consider the explanation on it.**

[Response]

Thank you very much for your highlighting. Accordingly, we have added a brief description of the convalescent rehabilitation ward to the Methods section.

“The convalescent rehabilitation ward is a ward for the intensive rehabilitation of patients who require ADLs assistance—including those with hip fracture or cerebrovascular disease—to prevent bedridden patients from staying bedridden, and help them return to their homes by improving their ADLs [42].” (page 9, lines 166–170)

10. **Page 8, line 178: Please consider to add some description for the demographics of the population such as age and sex at least.**

[Response]

We have added descriptions of the population's demographics, including age and sex distributions, to provide a clearer understanding of the study participants.

“The study included 8,109 participants (Fig 1). The median age of the participants was 86 years, with an interquartile range (IQR) of 82–91 years. Of the 8,108 subjects analyzed, 6,736 (83.1%) were women.” (pages 10–11, lines 194–197)

11. **Table S6 and Table 1 can be combined and shown as a Table 1 because authors are conducting a regression.**

[Response]

Thank you for pointing this out. We have combined Table S6 and Table 1 into a single Table 1 to enhance reader understanding. Additionally, we have renumbered the former Table S7 to Table S6: “S6 Table. Status of continuing rehabilitation for six months after surgery, stratified by sex and age groups (n=8,108).”

12. **Figure 2 should be added the value at each month and what is month means at x-axis.**

[Response]

Thank you very much for your comment. We have revised Fig 2 and S1 Fig to include the values for each month and clarified that the x-axis represents the months after surgery. The updated figure now clearly shows the implementation status of rehabilitation from the first month to the sixth month after surgery, providing detailed monthly data.

13. **Table 2: I’m not sure that the results of univariable analysis is needed because authors did not seem to mention them.**

[Response]

Thank you for your valuable comments. We believe that presenting both univariate and multivariate results will provide readers with a more comprehensive understanding of the data. The univariate results demonstrated a simple association between each predictor and the outcome, whereas the multivariate ones showed associations after controlling for all other predictors. However, we acknowledge that we have not discussed the results of the univariate analysis in the manuscript. If you feel that the univariate results are unnecessary, we would certainly be willing to remove them from Table 2.

14. **Table 2: Who has a responsibility for continuing rehabilitation after hip fracture surgery in Japan? At the acute or sub-acute moments, physicians in hospitals could order it. However, how about in the situation after several months in nursing facilities? Do physicians in clinics do it? Or can care managers in the long-term care insurance system do it?**

[Response]

Thank you for your comments. In Japan, rehabilitation under medical insurance is provided based on a physician’s prescription. Under long-term care insurance, users request assistance from a care manager who creates a care plan for them. This plan includes an assessment of the user's situation, as well as that of their family, planning of the necessary services, and coordination with service providers.^4^ Rehabilitation is generally provided when the attending physician deems it is necessary and it meets the prescribed criteria.

4. Konishi T, Inokuchi H, Yasunaga H. Services in public long-term care insurance in Japan. Annals of Clinical Epidemiology. 2024;6: 1–4. doi:10.37737/ace.24001

15. **I am worry about the possibility of incorrect and biased estimates because of the nested structure of the population. Especially, if physicians order the rehabilitation, patients are correlated with physicians. Also, physicians are nested by hospitals. Therefore, I think authors should consider this structure in the regression, using the multilevel analysis or cluster SE, even though authors are exploring the related factors simply.**

[Response]

Thank you for your valuable advice on the population-nesting structure. As you have pointed out, we acknowledge the need to consider the relationship between hospitals and physicians’ situational rehabilitation practices. However, this database does not include information regarding physicians who prescribe rehabilitation. Furthermore, it has not been compiled or maintained for use at the hospital or facility levels. Unfortunately, we could not conduct a multi-level analysis or other analyses that considered the nested structure of the population. The points you raised are critical, and we have added the following statement as a limitation and future perspective for the study:

“Second, this database does not contain information about the physicians who prescribed rehabilitation and information concerning the hospitals or facilities where the patients stayed is not well organized. Therefore, this study could not clarify the relationship between hospitals, facilities, and physicians with the implementation of rehabilitation. Further research is warranted to explore the perspectives of patients and their families on this matter [61,62], as well as facility-level [63] and regional-level factors [64].” (age 21, lines 337–343)

Thank you again for your valuable advice.

16. **Page 13, lines 219 – 229: Authors mentioned the issue around the transition from hospitals to other facilities as a possible reason of the decreasing proportion of rehabilitation over time after the operation. However, it seems to me there are other possible reasons. For example, some patients may be more likely to recover quickly. Other patients may not prefer to continue rehabilitation for whatever reason. Or perhaps there are limited resources for rehabilitation in the home or nursing home. This is a primary outcome in the study, so the authors should consider various possibilities.**

[Response]

As you pointed out, we also believe that it is necessary to consider other factors that may influence the implementation of rehabilitation. Thank you for your valuable suggestion. We have added the following sentences to address this question:

“Other factors should also be considered when attempting to explain the decline in the rehabilitation implementation rate over time after surgery. First, some patients may have experienced relatively rapid recovery in terms of ADL and physical function following hip fracture surgery, and thus no longer required rehabilitation. Second, some patients may have been reluctant to continue rehabilitation for various reasons. A previous study reported that feelings of vulnerability in patients with hip fractures persist until their function and activities are sufficiently restored to allow them to participate in meaningful activities [45]. Patients who feel burdened or lack confidence in their recovery are more likely to refuse rehabilitation. Third, some patients may have prioritized services other than rehabilitation. A population-based retrospective cohort study showed that older adults with dementia used home care services more often after sustaining hip fractures, compared to their counterparts without dementia [46]. Additionally, it is necessary to consider the possibility that rehabilitation resources are limited to the patients' residential areas. A study using the National Administrative Database of Canada compared the discharge destinations of patients with hip fractures from acute care hospitals by province, and reported significant variations in discharge destinations across them [47]. Our study could not elucidate the reasons for the decline in the rehabilitation implementation rate over time after surgery. However, future research should consider patient conditions, preferences, and available rehabilitation resources.” (pages 17–18, lines 246–265)

Additionally, we have included the following as a limitation of our study:

“This study was subject to several key limitations worth noting. First, we did not assess patient status—including physical function and ADL, as well as knowledge and attitudes in patients and their families—or the post-discharge environment. Some patients may have discontinued rehabilitation after surgery because their physical function and abilities to perform ADLs improved sufficiently. Second, this database does not contain information about the physicians who prescribed rehabilitation and information concerning the hospitals or facilities where the patients stayed is not well organized. Therefore, this study could not clarify the relationship between hospitals, facilities, and physicians with the implementation of rehabilitation. Further research is warranted to explore the perspectives of patients and their families on this matter [61,62], as well as facility-level [63] and regional-level factors [64].” (page 21, lines 333–343)

17. **Page 17, lines 313 - 315: Please clarify the directions of the association. (i.e., positive or negative)**

[Response]

We have revised the manuscript to clarify the direction of the association by adding the word "positively." The revised sentence now reads: “This study suggests that patients with hip fractures may discontinue rehabilitation after discharge from acute care hospitals. Factors such as older age, male sex, frailty, care dependency before hip fracture surgeries, and non-dementia might be positively associated with completing six months of postoperative rehabilitation.” (page 22, line 363).

Additionally, the word “actively” in the abstract as well.

”The following variables were positively associated with continuing rehabilitation for six months:” (page 3, lines 13)

**Minor comments:**

1. **Page 2, Line 43: an intermediate of what?**

[Response]

“An intermediate” indicates intermediate frailty risk as estimated using the hospital's frailty risk score. Therefore, we have revised “an intermediate” to “an intermediate frailty risk”. Thank you very much for pointing this out. (page 3, lines 14–15)

2. **Page 2, Line 45: Please consider to add some odds ratios for care dependency.**

[Response]

Thank you for pointing this out. In order to provide clearer information to our readers, we added odds ratios for care dependency.

[Detailed revision]

The following variables were positively associated with continuing rehabilitation for six months: males (adjusted odds ratio: 1.41 [95% confidence interval: 1.23–1.62]), an intermediate frailty risk (1.50 [1.24–1.82]), high frailty risk (2.09 [1.69–2.58]) estimated using the hospital frailty risk scores, and preoperative care dependency levels: support level 1 (1.69 [1.28–2.23]), support level 2 (2.34 [1.88–2.90]), care-need level 1 (2.04 [1.68–2.49]), care-need level 2 (2.42 [2.04–2.89]), care-need level 3 (1.45 [1.19–1.76]), care-need level 4 (1.40 [1.12–1.75]), and care-need level 5 (1.31 [0.93–1.85]). (page 3, lines 12–20)

3. **Table S2 is titled as S3.**

[Response]

We apologize for the incomplete description and have corrected the title of Table S2 from S3 to S2.

[Before]

S3 Table. The list of definitions of rehabilitation in medical insurance

[Detailed revision]

**S2 Table. The list of definitions of rehabilitation in medical insurance**
